# Volatile Compositions and Antifungal Activities of Native American Medicinal Plants: Focus on the Asteraceae

**DOI:** 10.3390/plants9010126

**Published:** 2020-01-19

**Authors:** Sims K. Lawson, Layla G. Sharp, Chelsea N. Powers, Robert L. McFeeters, Prabodh Satyal, William N. Setzer

**Affiliations:** 1 Department of Chemistry, University of Alabama in Huntsville, Huntsville, AL 35899, USA; skl0003@uah.edu (S.K.L.); lgs0003@uah.edu (L.G.S.); cnp0007@uah.edu (C.N.P.); rlm0004@uah.edu (R.L.M.); 2 Aromatic Plant Research Center, 230 N 1200 E, Suite 100, Lehi, UT 84043, USA; psatyal@aromaticplant.org

**Keywords:** ethnopharmacology, essential oil, chemical composition, *Cryptococcus neoformans*, cyclocolorenone

## Abstract

In the past, Native Americans of North America had an abundant traditional herbal legacy for treating illnesses, disorders, and wounds. Unfortunately, much of the ethnopharmacological knowledge of North American Indians has been lost due to population destruction and displacement from their native lands by European-based settlers. However, there are some sources of Native American ethnobotany remaining. In this work, we have consulted the ethnobotanical literature for members of the Asteraceae used in Cherokee and other Native American traditional medicines that are native to the southeastern United States. The aerial parts of *Eupatorium serotinum*, *Eurybia macrophylla*, *Eutrochium purpureum*, *Polymnia canadensis*, *Rudbeckia laciniata*, *Silphium integrifolium*, *Smallanthus uvedalia*, *Solidago altissima*, and *Xanthium strumarium* were collected from wild-growing plants in north Alabama. The plants were hydrodistilled to obtain the essential oils and the chemical compositions of the essential oils were determined by gas chromatography–mass spectrometry. The essential oils were tested for in-vitro antifungal activity against *Aspergillus niger*, *Candida albicans*, and *Cryptococcus neoformans*. The essential oil of *E. serotinum* showed noteworthy activity against *C. neoformans* with a minimum inhibitory concentration (MIC) value of 78 μg/mL, which can be attributed to the high concentration of cyclocolorenone in the essential oil.

## 1. Introduction

Many aspects of modern medicine have relied on the traditional knowledge of native cultures, including, for example, traditional Indian medicine (Ayurveda) [1], traditional Chinese medicine (TCM) [2], and traditional Islamic medicine [3]. Unfortunately, many of the traditional uses of medicinal plants are being lost due to several reasons. Recent generations are less interested in traditional knowledge, and habitat destruction and forced migration have reduced access to medicinal plants. The Native Americans of North America also had rich traditions of medicinal plant use. However, much of this knowledge has been lost due to population declines and displacement from native lands. Nevertheless, there are still some existing references to the ethnobotanical uses of medicinal plants by Native Americans [4].

*Eupatorium serotinum* Michx., “late boneset”, is native to eastern North America and ranges from Texas, Oklahoma, and Kansas, to the Atlantic coast and from the Gulf of Mexico north to Wisconsin and Michigan [5]. The Houma people of Louisiana used a decoction of the flowers to treat typhoid fever [6]. Extracts of the aerial parts of *E. serotinum* have yielded germacranolide sesquiterpenoids [7,8].

*Eurybia macrophylla* (L.) Cass. (syn. *Aster macrophyllus* L.), “bigleaf aster”, is native to southeastern Canada and northeastern United States, as far south as north Alabama and north Georgia [9]. The Iroquois used a decoction of the roots as a blood medicine and to treat venereal disease; the Ojibwa people ate the leaves of *E. macrophylla* as a medicine and food [6].

*Eutrochium purpureum* (L.) E.E. Lamont (syn. *Eupatorium purpureum* L.), “purple Joe-Pye weed”, ranges from central to eastern North America, from the Great Lakes region south to the Gulf of Mexico [10]. The Cherokee used the roots as a treatment for rheumatism, for kidney problems, and for “female problems”; the Chippewa inhaled the vapors from an infusion of the plant for colds; the Navajo used the plant as an antidote for poison; and the Potawatomi people applied a poultice of the leaves on burns [6].

*Polymnia canadensis* L., “white flower leafcup”, is found in eastern North America from Alabama and Georgia north to Ontario, and from Kansas and Oklahoma east to the Appalachians and New York [11]. The Houma people applied a poultice of the crushed leaves to swellings; the Iroquois used the plant to relieve toothache [6]. Extracts of the aerial parts of *P. canadensis* have yielded diterpenoid carboxylic acids and germacranolides [12].

*Rudbeckia laciniata* L., “cutleaf coneflower”, is widespread in the United States and Canada [13]. There are eight varieties of *R. laciniata*, namely *ampla*, *bipinnata*, *digitata*, *gaspereauensis*, *heterophylla*, *hortensia*, *humilis*, and *laciniata* [14]; *R. laciniata* var. *laciniata* is the common variety found in eastern North America [15]. The Cherokee ate the cooked greens to “keep well”, while the Chippewa applied a poultice of the flowers to treat burns [6]. Several lignans, flavonoid glycosides, and quinic acid derivatives have been isolated from the aerial parts of *R. laciniata*, and sesquiterpenoids have been isolated from root extracts [4].

*Silphium integrifolium* Michx., “whole-leaf rosinweed”, ranges from Wisconsin and Michigan, south through Alabama and Mississippi, and west as far as New Mexico [16]. An infusion of the leaves of *S. integrifolium* was taken by the Meskwaki people for “bladder troubles” [6]. Flavonoids, oleanolic acid glycosides, and phenolic acids have been identified in the aerial parts of *S. integrifolium* [17].

*Smallanthus uvedalia* (L.) Mack. (syn. *Polymnia uvedalia* (L.) L.), “yellow flower leafcup”, is found in the southeastern United States from Virginia to Florida, west to eastern Texas and Oklahoma [18]. The Cherokee used the bruised roots on burns and cuts; the Iroquois took an infusion of the shoots and roots to treat back pain and vomiting [6]. The plant is the source of several germacranolide sesquiterpenoids and *ent*-kaurane diterpenoids [4].

*Solidago altissima* L. (syn. *Solidago canadensis* L.), “Canada goldenrod”, ranges across most of North America from Canada to northern Mexico [19]. The Okanagan-Colville and the Thompson tribes used an infusion of the roots and shoots of *S. altissima* to treat fevers [6], and the Cherokee took an infusion of *Solidago* spp. to treat fevers. The phytochemistry of *S. canadensis* has been extensively studied and found to contain saponins [20,21], flavonoids [22,23,24], polyacetylenes [25,26], diterpenoids [27], and triterpenoids [28].

*Xanthium strumarium* L., “rough cocklebur”, ranges throughout North America and is considered a noxious weed in the southeastern United States [29]. The White Mountain Apache tribe took a root decoction to treat fevers; the Mahuma people of Southern California used the plant to treat rheumatism, tuberculosis, and gonorrhea [6]. The aerial parts of *X. strumarium* contain alkaloids, sesquiterpene lactones (guaianolides, germacranolides, and elemanolides), phenolic compounds, and the toxic carboxylic acid atractyloside, a kaurene glycoside [30].

We have had an interest in the volatile chemistry and biological activity of Native American medicinal plants [31,32,33,34,35,36,37,38,39,40,41,42], including members of the Asteraceae [43,44,45]. As part of our continuing investigations, the purpose of this work was to seek out additional species of Asteraceae important in Native American traditional medicine growing wild in northern Alabama and to obtain the essential oils by hydrodistillation of the aerial parts. As a test for biological activity, the essential oils were then screened for antifungal activity against three potentially pathogenic fungal strains. *Aspergillus niger*, *Candida albicans*, and *Cryptococcus neoformans* are the causative agents of opportunistic *Aspergillus* lung disease, candidiasis, and cryptococcosis, respectively.

## 2. Results and Discussion

The essential oils from *E. serotinum*, *E. macrophylla*, *E. purpureum*, *P. canadensis*, *R. laciniata*, *S. integrifolium*, *S. uvedalia*, *S. altissima*, and *X. strumarium* were obtained from the fresh aerial parts of the plants by hydrodistillation, generally in low yield. The essential oils were analyzed by GC and GC–MS (Tables 1, 3–9, and 11).

### 2.1. Eupatorium serotinum Michx.

The essential oil from the aerial parts of *E. serotinum* was rich in sesquiterpenoids, with cyclocolorenone (23.38%), germacrene D (6.58%), and palustrol (5.32%), along with an unidentified sesquiterpenoid (5.72%) as the major components (Table 1).

To our knowledge, there have been no previous reports on the essential oil composition of *E. serotinum*. The phytochemistry of the genus *Eupatorium* has been reviewed [46] and there have been numerous reports on the essential oil compositions from other species of the genus (Table 2). There is much variability in the essential oil compositions of *Eupatorium* species, both between species and within species. Nevertheless, sesquiterpenoids often dominate the essential oils of *Eupatorium* species.

### 2.2. Eurybia macrophylla (L.) Cass.

Monoterpene hydrocarbons, limonene (28.66%), β-pinene (8.57%), and terpinolene (5.35%), and germacrane sesquiterpenes, germacrene D (19.81%), and germacrene B (7.07%), were the major components in the essential oil of *E. macrophylla* (Table 3). To our knowledge, there are no reports on essential oil compositions of any *Eurybia* species.

### 2.3. Eutrochium purpureum (L.) E.E. Lamont (syn. Eupatorium purpureum L.)

The major components in the essential oil of *E. purpureum* were the green leaf volatiles (2*E*)-hexenal (60.59%) and hexanal (6.78%), along with the aromatic compounds eugenol (11.68%) and methyl salicylate (10.31%; Table 4). There have apparently been no previous reports on the essential oil composition of *E. purpureum* or any other *Eutrochium* species. There are numerous reports on *Eupatorium* essential oils, however (see above).

### 2.4. Polymnia canadensis L.

α-Phellandrene (28.30%), α-pinene (19.71%), and germacrene D (11.42%) were the major components in the essential oil from the aerial parts of *P. canadensis* (Table 5). The volatile chemical profile of *P. canadensis* in this current work is in marked contrast to our previous report on this species [45]. Previous samples were rich in the sesquiterpene hydrocarbons germacrene D (63.6% and 44.5%) and β-caryophyllene (15.9% and 14.8%). The differences in compositions are likely due to seasonal variation (the current sample was collected in July, 2018, while the previous samples were collected in September, 2015, and December, 2016, respectively). We cannot rule out, however, chemical profile differences attributable to environmental differences or biotic differences (e.g., genetics, herbivory, or pathogen stress).

### 2.5. Rudbeckia laciniata L.

Monoterpene hydrocarbons dominated the essential oil of *R. laciniata* (Table 6). The major components were limonene (58.07%), α-pinene (10.18%), β-pinene (9.21%), and myrcene (5.26%). While *R. laciniata* essential oil was rich in monoterpene hydrocarbons, the essential oils of *R. fulgida* and *R. hirta* were rich in sesquiterpene hydrocarbons [43]. The major components in *R. fulgida* essential oil were germacrene D (30.1%), δ-cadinene (17.8%), β-caryophyllene (10.0%), and γ-muurolene (8.9%), along with (*E*)-β-ocimene (6.2%) and (2*E*)-hexenal (6.0%). Similarly, the major components of *R. hirta* essential oil were germacrene D (23.6%), δ-cadinene (16.2%), β-caryophyllene (4.7%), γ-muurolene (8.1%), as well as (*E*)-β-ocimene (15.2%) and (2*E*)-hexenal (20.2%) [43]. The leaf essential oil of *Rudbeckia triloba*, collected in Bucharest, Romania, was rich in monoterpene hydrocarbons, α-pinene (46.0%), sabinene (9.6%), and β-phellandrene (24.6%), along with germacrene D (6.1%), but devoid of limonene [73]. Thus, there do not seem to be any consistent chemical markers for the *Rudbeckia* genus.

### 2.6. Silphium integrifolium Michx.

The major components in the essential oil from the aerial parts of *S. integrifolium* were α-pinene (58.59%) and β-pinene (14.69%), followed by myrcene (9.70%; Table 7). Kowalski has extensively examined the essential oils of *S. integrifolium* as well as *S. trifoliatum* cultivated in Poland [74,75,76,77,78]. The leaf essential oil of *S. integrifolium* from Poland had α-pinene (7.3–9.8%), germacrene D (4.0–28.4%), *allo*-aromadendrene (3.7–8.5%), caryophyllene oxide (6.1–12.4%), and silphiperfol-6-en-5-one (3.7–5.1%) [74,75]; while the floral essential oil was made up of α-pinene (13.4–14.0%), camphene (5.3–5.7%), *trans*-verbenol (5.2–6.3%), bornyl acetate (6.5–7.0%), and *allo*-aromadendrene (5.6–6.1%) [74,77]. Thus, there are major qualitative and quantitative differences between the samples from Alabama and from Poland.

### 2.7. Smallanthus uvedalia (L.) Mack.

Monoterpene hydrocarbons dominated the essential oil of *S. uvedalia* (Table 8). α-Pinene (62.56%) was the major component, followed by limonene (11.43%) and β-pinene (6.00%). The chemical composition of this monoterpene-rich essential oil is very different from the compositions collected previously by us [45]. The previous samples, collected in February 2016, were dominated by β-caryophyllene (24.5% and 16.5%) and caryophyllene oxide (19.8% and 14.2%). The sample of *S. uvedalia* in this present work was collected in September 2018. The differences in composition may be due to seasonal variation, genetic differences, or environmental stresses. Nevertheless, α-pinene has dominated the essential oil compositions of other *Smallanthus* species. For example, α-pinene was the major component in the essential oil of *S. maculatus* from Costa Rica (32.9% α-pinene), which was also rich in camphene (5.4%), β-pinene (7.1%), β-caryophyllene (10.7%), germacrene D (13.7%), and bicyclogermacrene (6.6%) [79]. Likewise, the essential oil of *S. quichensis* from Costa Rica was also dominated by α-pinene (35.5–64.5%) with lesser concentrations of α-phellandrene (0.1–9.0%), *p*-cymene (0.1–11.5%), limonene (2.1–5.8%), β-phellandrene (up to 9.2%), and 1,8-cineole (up to 9.7%) [80]. In contrast, *S. sonchifolia* essential oil, grown in Sichuan, China, was made up of β-phellandrene (26.3%), β-bourbonene (10.2%), β-caryophyllene (14.0%), and β-cubebene (17.6%) [81].

### 2.8. Solidago altissima L. (syn. Solidago canadensis L.)

The major components in the essential oil from the aerial parts of *S. altissima* (syn. *S. canadensis*) from Alabama were α-pinene (13.91%), sabinene (14.25%), myrcene (20.29%), bornyl acetate (14.44%), and germacrene D (10.67%; Table 9). Previous examinations of *S. canadensis* essential oils have shown germacrene D to be one of the most abundant components (Table 10). However, Weyerstahl and co-workers [82] found curlone (23.5%) to be a major component of *S. canadensis* from Poland, Schmidt and co-workers [83] found cyclocolorenone (38%) to be a major component in *S. canadensis* from northern Germany, and Kasali and co-workers [84] found 6-*epi*-β-cubebene to be a major component (20.5%) in *S. canadensis* essential oil from Poland. Interestingly, none of these compounds was detected in the sample of *S. altissima* essential oil from Alabama.

### 2.9. Xanthium strumarium L.

The major volatile components from the aerial parts of *X. strumarium* were limonene (48.23%), myrcene (14.31%), germacrene D (13.92%), (2*E*)-hexenal (5.79%), and sabinene (4.89%; Table 11). The compositions of *Xanthium strumarium* essential oils from the Middle East have been reported, including Iran [93,94] and Pakistan [95]. The leaf essential oil from Khoramabad, Iran, was composed largely of limonene (24.7%), borneol (10.6%), bornyl acetate (5.9%), and β-cubebene (6.3%) [93]. The leaf essential oil from Zabol, Iran, was qualitatively similar, limonene (20.3%), borneol (11.6%), bornyl acetate (4.5%), and β-cubebene (3.8%), but also contained a large concentration of *cis*-β-guaiene (34.2%), which was not observed in any other *X. strumarium* essential oils [94]. The leaf essential oil of *X. strumarium* from Lahore, Pakistan, contained limonene (5.7%), β-caryophyllene (17.5%), spathulenol (6.1%), and α-cadinol (6.7%) as major components [95]. The differences in chemical compositions may be related to different genetic factors as well as geographical location; Tropicos^®^ currently lists 13 subordinate taxa for *X. strumarium* [14].

### 2.10. Antifungal Screening

Depending on material available, the essential oils were screened for antifungal activity against the opportunistic fungal pathogens *Aspergillus niger*, *Candida albicans*, and *Cryptococcus neoformans* using the microbroth dilution technique (Table 12). The essential oil of *E. serotinum* showed promising antifungal activity against *C. neoformans* with a minimum inhibitory concentration (MIC) value of 78 μg/mL. The high concentration of cyclocolorenone in *E. serotinum* is likely responsible for the observed antifungal activity of this essential oil. Cyclocolorenone had been previously reported to show antifungal activity against *Curvularia lunata*, *Chaetomium cochliodes*, and *Chaetomium spinusum* [96]. Germacrene D may also contribute to the antifungal activity of *E. serotinum* essential oil as well as essential oils of *E. macrophylla*, *P. canadensis*, and *R. laciniata*. Germacrene D has shown antifungal activity against *Aspergillus niger* with MIC of 39 μg/mL [97].

The modest antifungal activity of *E. purpureum* is somewhat surprising. The major components were hexanal, (2*E*)-hexenal, methyl salicylate, and eugenol. Hexanal [98] and (2*E*)-hexenal [99,100] are both known to be antifungal to plant pathogenic fungi. Methyl salicylate is only weakly antifungal against *A. niger*, *C. albicans*, or *C. neoformans*, but eugenol is somewhat active (see Table 13). Monoterpene hydrocarbons such as α-pinene, β-pinene, limonene, or myrcene show only weak antifungal activity (Table 13) and are not expected to contribute to the antifungal activities of the essential oils unless there are synergistic effects of these components (see, for example [101,102]). The mechanisms of antifungal activity of essential oils are poorly understood. However, it has been suggested that essential oils and their components, being lipophilic, can disrupt the membranes of fungi causing membrane permeability [103].

## 3. Materials and Methods

### 3.1. Plant Material

Aerial parts of each plant were collected from various sites in north Alabama (Table 14). Plants were identified by S.K. Lawson and voucher specimens were deposited in the University of Alabama in Huntsville herbarium (HALA). The fresh plant material (aerial parts) were chopped and hydrodistilled using a Likens–Nickerson apparatus with continuous extraction with CH_2_Cl_2_ for three hours. The solvent was evaporated to give pale yellow essential oils (Table 14).

### 3.2. Gas Chromatography–Mass Spectrometry

The Asteraceae essential oils were analyzed by GC–MS using a Shimadzu GC–MS-QP2010 Ultra fitted with a Phenomenex ZB-5ms column as previously described [104]. Identification of the essential oil components was determined by comparison of their retention indices, determined with respect to a homologous series of *n*-alkanes and their mass spectral fragmentation patters with those from available databases (Adams [105], NIST17 [106], and FFNSC 3 [107]) or in our in-house library [108].

### 3.3. Gas Chromatography–Flame Ionization Detection

Quantification of the essential oils was determined by GC–FID using a Shimadzu GC 2010 instrument fitted with a ZB-5 column [104], using the same parameters that were used for the GC–MS. The concentrations (average of three measurements ± standard deviations) are based on peak integration without standardization.

### 3.4. Antifungal Screening

The essential oils were screened for antifungal activity against *Aspergillus niger* (ATCC 16888), *Candida albicans* (ATCC 18804), and *Cryptococcus neoformans* (ATCC 24607) using the microbroth dilution method as previously described [109]. Amphotericin B was used as the positive control and RPMI medium was used as the negative control. The antifungal assays were carried out in triplicate.

## 4. Conclusions

There is much intraspecific variation in essential oil compositions of these members of the Asteraceae. Much of the variation can be attributed to geographical location or seasonal variation. *Eupatorium serotinum* essential oil showed notable antifungal activity against *Cryptococcus neoformans*. However, the yield of this essential oil (0.013%) is too low to be considered as pharmacologically useful. If suitable sources of the major component cyclocolorenone can be identified, then this compound may serve as important antifungal template for further elaboration.

## Figures and Tables

**Table 1:** Chemical composition of the essential oil of *Eupatorium serotinum* Michx.

RI ^a^	RI ^b^	Compound	% ± SD	RI ^a^	RI ^b^	Compound	% ± SD
802	801	Hexanal	0.16 ± 0.02	1531	1533	*trans*-Cadina-1,4-diene	0.20 ± 0.07
810	796	2-Hexanol	0.92 ± 0.01	1540	---	Unidentified ^e^	1.28 ± 0.05
850	846	(2*E*)-Hexenal	0.86 ± 0.11	1542	1539	α-Copaen-11-ol	7.89 ± 0.13
932	932	α-Pinene	0.17 ± 0.01	1548	---	Unidentified ^f^	1.76 ± 0.04
949	946	Camphene	1.78 ± 0.02	1550	---	Unidentified ^g^	0.75 ± 0.03
977	974	β-Pinene	0.18 ± 0.02	1558	1559	Germacrene B	0.44 ± 0.03
999	1001	δ-2-Carene	0.15 ± 0.01	1562	---	Eudesmenol ^h^	0.32 ± 0.09
1029	1024	Limonene	0.26 ± 0.01	1569	1567	Palustrol	5.32 ± 0.12
1283	1287	Bornyl acetate	4.72 ± 0.07	1575	1574	Germacra-1(10),5-dien-4β-ol	0.91 ± 1.10
1326	---	Unidentified ^c^	0.93 ± 0.03	1581	1577	Spathulenol	1.58 ± 0.83
1346	1345	α-Cubebene	0.59 ± 0.01	1588	1590	Globulol	0.58 ± 0.03
1375	1374	α-Copaene	0.11 ± 0.05	1593	1592	Viridiflorol	1.12 ± 0.09
1383	1387	β-Bourbonene	0.06 ± 0.01	1596	---	Unidentified ^i^	1.20 ± 0.02
1397	1387	β-Cubebene	3.65 ± 0.09	1603	1602	Ledol	2.80 ± 0.03
1406	1409	α-Gurjunene	0.74 ± 0.02	1620	1611	Germacra-1(10),5-dien-4α-ol	1.44 ± 0.13
1417	1419	β-Ylangene	0.09 ± 0.03	1622	1624	Selina-6-en-4β-ol	0.31 ± 0.03
1418	1417	β-Caryophyllene	0.96 ± 0.01	1627	1627	1-*epi*-Cubenol	0.61 ± 0.10
1428	1434	γ-Elemene	0.28 ± 0.05	1638	1639	*cis*-Guaia-3,9-dien-11-ol	0.12 ± 0.01
1448	1448	*cis*-Muurola-3,5-diene	0.07 ± 0.03	1642	1638	τ-Cadinol	0.80 ± 0.03
1455	1452	α-Humulene	0.41 ± 0.04	1642	1640	τ-Muurolol	0.62 ± 0.08
1471	1475	*trans*-Cadina-1(6),4-diene	0.25 ± 0.03	1646	1644	α-Muurolol (=δ-Cadinol)	0.69 ± 0.07
1480	1484	Germacrene D	6.58 ± 0.09	1648	1646	Agarospirol II	1.10 ± 0.04
1486	1488	δ-Selinene	0.27 ± 0.02	1654	1652	α-Cadinol	2.31 ± 0.04
1488	1489	β-Selinene	0.17 ± 0.01	1668	---	Unidentified^j^	5.72 ± 0.14
1491	1493	*trans*-Muurola-4(14),5-diene	0.69 ± 0.03	1751	1759	Cyclocolorenone	23.38 ± 0.43
1494	1493	*epi*-Cubebol	1.81 ± 0.03			Green leaf volatiles	1.94
1497	1500	α-Muurolene	0.34 ± 0.02			Monoterpene hydrocarbons	2.53
1502	---	Unidentified ^d^	1.30 ± 0.02			Oxygenated monoterpenoids	4.72
1512	1513	γ-Cadinene	0.21 ± 0.00			Sesquiterpene hydrocarbons	19.14
1514	1514	Cubebol	4.18 ± 0.11			Oxygenated sesquiterpenoids	57.90
1517	1522	δ-Cadinene	3.02 ± 0.25			Total Identified	86.23

^a^ RI = Retention index determined in reference to a homologous series of *n*-alkanes on a ZB-5ms column. ^b^ RI values from the databases. ^c^ MS(EI) (mass spectrum (electron impact)): 162(84%), 147(96%), 133(20%), 120(32%), 119(41%), 108(35%), 105(100%), 91(63%), 79(29%), 77(22%), 55(11%), 53(12%), 41(14%). ^d^ MS(EI): 202(7%), 187(9%), 162(68%), 159(31%), 147(50%), 145(32%), 132(49%), 119(66%), 105(89%), 91(48%), 81(18%), 79(20%), 77(16%), 59(100%), 43(20%), 41(20%). ^e^ MS(EI): 202(4%), 187(13%), 162(56%), 159(59%), 147(39%), 145(40%), 132(73%), 131(39%), 119(73%), 106(48%), 105(88%), 91(47%), 81(16%), 79(25%), 77(19%), 59(100%), 55(18%), 43(19%), 41(20%). ^f^ MS(EI): 220(24%), 205(17%), 163(19%), 120(35%), 110(100%), 105(20%), 95(35%), 69(44%), 55(20%), 41(24%). ^g^ MS(EI): 220(47%), 163(25%), 161(32%), 121(100%), 108(42%), 93(42%), 81(59%), 69(17%), 55(15%), 41(18%). ^h^ Correct isomer not identified. ^i^ MS(EI): 220(49%), 205(7%), 163(33%), 161(28%), 121(100%), 108(40%), 93(35%), 81(80%), 69(20%), 55(17%), 41(19%). ^j^ MS(EI): 202(46%), 187(67%), 174(40%), 162(60%), 159(100%), 147(89%), 134(30%), 131(23%), 119(62%), 105(71%), 91(50%), 59(61%), 43(20%), 41(22%).

**Table 2:** Major components and biological activities of *Eupatorium* essential oils.

*Eupatorium* spp. Essential Oil	Location	Major Components	Biological Activity	Ref.
*E. adenophorum* (aerial parts)	Nainital, India	camphene (8.9%), *p*-cymene (16.6%), bornyl acetate (15.6%), amorph-4-en-7-ol (9.6%), α-cadinol (6.2%), amorpha-4,7(11)-dien-8-one (7.8%)	none reported	[47]
*E. adenophorum* (leaves)	Palampur, India	bornyl acetate (9.0%), germacrene D (5.7%), β-bisabolene (6.2%), 1-naphthalenol (17.5%), α-bisabolol (9.5%)	Antibacterial (*Rhodococcus rhodochrous*, MBC 12.5 μL/mL)	[48]
*E. adenophorum* (twigs)	Uttar Pradesh, India	camphene (12.1%), α-phellandrene (8.6%), α-terpinene (6.5%), *p*-cymene (11.6%), bornyl acetate (10.6%), acoradiene (10.1%), α-bisabolol (5.3%)	Antibacterial (*Erwinia herbicola*, MIC 0.25 μL/mL; *Pseudomonas putida*, MIC 2.0 μL/mL)	[49]
*E. adenophorum* (inflorescence)	Palampur, India	bornyl acetate (6.3%), β-caryophyllene (5.4%), γ-muurolene (11.7%), γ-curcumene (5.7%), γ-cadinene (18.4%), 3-acetoxyamorpha-4,7(11)-dien-8-one (7.4%)	Antifungal (*Macrophomina phaseolina*, EC_50_ 0.076 μL/mL; *Rhizoctonia solani*, EC_50_ 0.094 μL/mL; *Fusarium oxysporum*, EC_50_ 0.120 μL/mL)	[50]
*E. amygdalinum* (aerial parts)	Amapá, Brazil	β-cubebene (5.7%), β-caryophyllene (12.3%), germacrene D (15.5%), δ-cadinene (5.8%), caryophyllene oxide (17.4%)	none reported	[51]
*E. argentinum* (leaves)	Córdoba, Argentina	α-pinene (17.0%), β-pinene (6.1%), *p*-cymene (12.5%), thymyl acetate (9.7%), β-caryophyllene (7.2%)	none reported	[52]
*E. arnottianum* (aerial parts)	Córdoba, Argentina	α-pinene (13.7%), *p*-cymene (30.0%), β-ocimene (5.3%), thymyl acetate (12.3%), β-caryophyllene (11.7%)	none reported	[53]
*E. arnottianum* (aerial parts)	Córdoba, Argentina	limonene (32.7%), piperitenone (21.2%), *trans*-dihydrocarvone (10.2%), camphor (6.8%), *cis*-dihydrocarvone (6.7%)	Antiviral (HSV-1, IC_50_ 52.1 μg/mL; DENV-2, IC_50_ 38.2 μg/mL)	[54]
*E. arnottii* (aerial parts)	San Luis, Argentina	β-caryophyllene (7.9%), γ-elemene (5.9%), germacrene D (9.8%), cadinene (5.8%), spathulenol (10.6%), phytol (8.1%)	Insecticidal (*Tribolium castaneum*, ED_50_ 0.15 mg/cm2)	[55]
*E. ballotaefolium* (aerial parts)	Ceará, Brazil	α-pinene (6.2%), sabinene (6.5%), β-pinene (5.4%), myrcene (7.3%), limonene (15.3%), (*E*)-β-ocimene (10.5%), β-caryophyllene (7.5%)	none reported	[56]
*E. betonicaeforme* (leaves)	Ceará, Brazil	β-caryophyllene (36.1%), α-humulene (13.3%), γ-muurolene (20.3%), bicyclogermacrene (15.0%)	Larvicidal (*Aedes aegypti*, LC_50_ 129 μg/mL)	[57]
*E. buniifolium* (aerial parts)	Canelones, Uruguay	α-pinene (14.7%), β-elemene (12.2%), germacrene D (11.5%), trans-β-guaiene (6.5%)	none reported	[58]
*E. buniifolium* (aerial parts)	San Luis, Argentina	α-pinene (51.0%), sabinene (7.5%), limonene (9.6%), β-caryophyllene (5.2%)	Insecticidal (*Tribolium castaneum*, ED_50_ 0.15 mg/cm^2^)	[55]
*E. buniifolium* (leaves)	Canelones, Uruguay	α-pinene (8.2%), germacrene D (11.1%), trans-β-guaiene (7.4%)	Varroacide (*Varroa destructor*, LD_99_ 0.3 mg/mL)	[59]
*E. cannabinum* ssp. *cannabinum* (aerial parts)	Agerola, Italy	δ-2-carene (6.5%), germacrene D (33.5%), α-farnesene (12.9%)	Antibacterial (*Staphylococcus aureus*, *Streptococcus fecalis*, *Bacillus subtilis*, *Bacillus cereus*, MIC 1.25 mg/mL)	[60]
*E. cannabinum* (leaves)	Tuscany, Italy	thymol methyl ether (7.8%), germacrene D (29.2%), spathulenol (7.3%)	none reported	[61]
*E. cannabinum* ssp. *corsicum* (aerial parts)	Corsica, France	α-phellandrene (19.0%), *p*-cymene (5.2%), germacrene D (28.5%)	none reported	[62]
*E. cannabinum* (aerial parts)	Mazandaran, Iran	α-terpinene (17.8%), thymol methyl ether (5.2%), germacrene D (9.1%)	none reported	[63]
*E. cannabinum* (leaves)	Vilnius, Lithuania	thymol methyl ether (5.7%), neryl acetate (9.4%), germacrene D (11.3%), β-bisabolene (6.7%)	none reported	[64]
*E. capillifolium* (aerial parts)	Cuba	*p*-cymene (23.7%), thymol methyl ether (8.9%), β-bisabolene (8.2%), selin-11-en-4α-ol (12.3%)	none reported	[65]
*E. capillifolium* (aerial parts)	Mississippi, USA	myrcene (15.7%), α-phellandrene (6.5%), thymol methyl ether (36.3%), 2,5-dimethoxy-p-cymene (20.8%)	Insecticidal (*Stephanitis pyrioides*, LC_50_ 5800 μg/mL)	[66]
*E. catarium* (aerial parts)	Córdoba, Argentina	spathulenol (15.5%), β-caryophyllene (7.8%), germacrene D (5.5%), bicyclogermacrene (5.1%)	Antiviral (HSV-1, IC_50_ 47.9 μg/mL; DENV-2, IC_50_ 57.3 μg/mL)	[54]
*E. conyzoides* (aerial parts)	Tocantins, Brazil	β-caryophyllene (7.1%), α-humulene (6.6%), germacrene D (16.8%), bicyclogermacrene (7.2%), spathulenol (8.3%)	none reported	[51]
*E. glabratum* (leaves)	Michoacán, México	α-pinene (29.5%), β-pinene (6.3%), α-phellandrene (19.6%)	Insecticidal (*Sitophilus zeamais*, LC_50_ 18.0 μL/mL)	[67]
*E. hecatanthum* (leaves)	Córdoba, Argentina	α-pinene (13.4%), β-pinene (7.8%), β-ocimene (6.2%), carvacrol (7.1%), thymyl acetate (10.6%), β-caryophyllene (8.1%)	none reported	[52]
*E. inulaefolium* (aerial parts)	San Luis, Argentina	limonene (9.7%), δ-elemene (10.6%), β-caryophyllene (27.7%), α-humulene (5.9%), patchoulene (9.2%), germacrene D (13.7%), viridiflorol (9.2%)	Insecticidal (*Tribolium castaneum*, ED_50_ 0.15 mg/cm^2^)	[55]
*E. laevigatum* (aerial parts)	Roraima, Brazil	germacrene D (8.6%), selina-3,7(11)-diene (6.1%), spathulenol (5.4%), globulol (16.2%), laevigatin (23.6%)	none reported	[51]
*E. laevigatum* (leaves)	Rio Grande do Sul, Brazil	germacrene D (11.7%), bicyclogermacrene (9.3%), laevigatin (59.6%)	none reported	[68]
*E. macrophyllum* (aerial parts)	Chapada dos Guimarães, Brazil	sabinene (46.7%), limonene (23.3%)	none reported	[51]
*E. marginatum* (aerial parts	Ananindeua, Pará, Brazil	*ar*-curcumene (6.8%), α-zingiberene (57.5%), β-sesquiphellandrene (7.1%), (*E*)-γ-bisabolene (9.7%)	none reported	[51]
*E. marginatum* (aerial parts	Roraima, Brazil	α-gurjunene (19.5%), germacrene D (14.8%), α-selinene (9.0%), (E)-γ-bisabolene (5.0%)	none reported	[51]
*E. odoratum* (aerial parts)	Thitsanulok, Thailand	α-pinene (8.4%), β-pinene (5.6%), pregeijerene (17.6%), germacrene D (11.1%), β-caryophyllene (7.3%), vestitenone (6.5%)	none reported	[69]
*E. odoratum* (leaves)	Lagos, Nigeria	α-pinene (42.2%), β-pinene (10.6%), β-caryophyllene (5.4%), germacrene D (9.7%), β-copaen-4α-ol (9.4%)	Antibacterial (*Bacillus cereus*, MIC 39 μg/mL), antifungal (*Aspergillus niger*, MIC 78 μg/mL)	[70]
*E. odoratum* (aerial parts)	Western Ghats, India	*cis*-sabinene hydrate (5.7%), pregeijerene (14.2%), *epi*-cubebol (9.8%), cubebol (8.6%)	none reported	[71]
*E. squalidum* (aerial parts)	Amapá, Brazil	β-caryophyllene (6.2%), germacrene D (21.6%), bicyclogermacrene (6.0%), spathulenol (14.2%), globulol (25.1%)	none reported	[51]
*E. squalidum* (aerial parts)	Tocantins, Brazil	limonene (6.6%), β-caryophyllene (9.6%), germacrene D (10.4%), caryophyllene oxide (30.1%)	none reported	[51]
*E. subhastatum* (leaves)	Córdoba, Argentina	α-pinene (11.0%), β-pinene (5.9%), *p*-cymene (24.8%), α-copaene (5.1%), α-humulene (5.1%)	none reported	[52]
*E. triplinerve* (leaves)	Lucknow, India	δ-elemene (5.9%), β-caryophyllene (14.7%), selina-4(15),7(11)-dien-8-one	none reported	[72]
*E. viscidum* (aerial parts)	San Luis, Argentina	6-methyl-5-hepten-2-one (18.2%), spathulenol (25.2%)	Insecticidal (*Tribolium castaneum*, ED_50_ > 0.212 mg/cm^2^)	[55]

**Table 3:** Chemical composition of the essential oil of *Eurybia macrophylla* (L.) Cass.

RI ^a^	RI ^b^	Compound	% ± SD	RI ^a^	RI ^b^	Compound	% ± SD
801	797	(3*Z*)-Hexenal	0.06 ± 0.01	1387	1389	β-Elemene	1.48 ± 0.04
802	801	Hexanal	0.31 ± 0.05	1418	1417	β-Caryophyllene	4.60 ± 0.07
850	846	(2*E*)-Hexenal	1.44 ± 0.06	1427	1434	γ-Elemene	3.16 ± 0.01
865	863	1-Hexanol	0.11 ± 0.02	1431	1432	*trans*-α-Bergamotene	0.05 ± 0.02
924	924	α-Thujene	0.16 ± 0.01	1454	1452	α-Humulene	0.64 ± 0.01
932	974	α-Pinene	3.12 ± 0.04	1473	1471	Massoia lactone	0.35 ± 0.04
948	946	Camphene	0.60 ± 0.00	1480	1484	Germacrene D	19.81 ± 0.20
971	969	Sabinene	0.15 ± 0.02	1487	1489	β-Selinene	0.31 ± 0.05
977	974	β-Pinene	8.57 ± 0.07	1494	1500	Bicyclogermacrene	1.95 ± 0.02
988	988	Myrcene	1.79 ± 0.02	1497	1500	α-Muurolene	0.16 ± 0.03
989	988	Dehydro-1,8-cineole	0.28 ± 0.01	1516	1522	δ-Cadinene	0.19 ± 0.02
1006	1002	α-Phellandrene	0.88 ± 0.01	1536	1537	α-Cadinene	0.10 ± 0.02
1016	1014	α-Terpinene	0.16 ± 0.02	1557	1559	Germacrene B	7.07 ± 0.07
1024	1020	*p*-Cymene	0.15 ± 0.01	1575	1577	Spathulenol	0.10 ± 0.01
1028	1024	Limonene	28.66 ± 0.33	1581	1582	Caryophyllene oxide	0.38 ± 0.01
1030	1025	β-Phellandrene	0.75 ± 0.03	1595	1592	Viridiflorol	0.23 ± 0.03
1034	1032	(*Z*)-β-Ocimene	0.18 ± 0.00	1627	1629	*iso*-Spathulenol	0.08 ± 0.01
1044	1044	(*E*)-β-Ocimene	2.14 ± 0.02	1641	1638	τ-Cadinol	0.09 ± 0.02
1057	1054	γ-Terpinene	0.37 ± 0.01	1643	1640	τ-Murrolol	0.12 ± 0.03
1084	1086	Terpinolene	5.35 ± 0.08	1646	1644	α-Muurolol (=δ-Cadinol)	0.10 ± 0.01
1112	1114	(*E*)-4,8-Dimethylnona-1,3,7-triene	0.35 ± 0.01	1654	1652	α-Cadinol	0.47 ± 0.02
1124	1118	*cis-p*-Menth-2-en-1-ol	0.72 ± 0.01	1832	1835	Neophytadiene	0.05 ± 0.02
1142	1136	*trans-p*-Menth-2-en-1-ol	0.44 ± 0.01	1838	1841	Phytone	0.05 ± 0.02
1187	1179	*p*-Cymen-8-ol	0.21 ± 0.03			Green leaf volatiles	1.91
1195	1186	α-Terpineol	0.06 ± 0.02			Monoterpene hydrocarbons	53.03
1197	1195	*cis*-Piperitol	0.15 ± 0.02			Oxygenated monoterpenoids	2.30
1209	1207	*trans*-Piperitol	0.17 ± 0.01			Sesquiterpene hydrocarbons	40.03
1283	1287	Bornyl acetate	0.27 ± 0.10			Oxygenated sesquiterpenoids	1.59
1292	1293	Undecan-2-one	0.05 ± 0.01			Diterpenoids	0.11
1333	1335	δ-Elemene	0.50 ± 0.00			Others	0.75
						Total Identified	99.72

^a^ RI = Retention index determined in reference to a homologous series of *n*-alkanes on a ZB-5ms column. ^b^ RI values from the databases.

**Table 4:** Chemical composition of the essential oil of *Eutrochium purpureum* (L.) E.E. Lamont.

RI ^a^	RI ^b^	Compound	% ± SD	RI ^a^	RI ^b^	Compound	% ± SD
801	797	(3*Z*)-Hexenal	1.01 ± 0.11	1206	1201	Decanal	0.37 ± 0.05
802	801	Hexanal	6.78 ± 0.17	1351	1356	Eugenol	11.68 ± 0.14
850	946	(2*E*)-Hexenal	60.59 ± 1.00	1417	1417	β-Caryophyllene	0.24 ± 0.02
865	963	1-Hexanol	2.35 ± 0.41	1479	1484	Germacrene D	0.67 ± 0.10
931	932	α-Pinene	1.48 ± 0.09	1559	1561	(*E*)-Nerolidol	0.50 ± 0.01
943	---	Unidentified ^c^	0.56 ± 0.07			Green leaf volatiles	71.47
1004	998	Octanal	0.33 ± 0.04			Monoterpene hydrocarbons	2.36
1005	1004	(3*Z*)-Hexenyl acetate	0.72 ± 0.12			Sesquiterpene hydrocarbons	0.91
1028	1024	Limonene	0.88 ± 0.07			Oxygenated sesquiterpenoids	0.50
1045	1036	Benzene acetaldehyde	0.60 ± 0.03			Benzenoids	22.59
1105	1100	Nonanal	0.91 ± 0.19			Fatty aldehydes	1.61
1192	1190	Methyl salicylate	10.31 ± 0.18			Total Identified	99.44

^a^ RI = Retention index determined in reference to a homologous series of *n*-alkanes on a ZB-5ms column. ^b^ RI values from the databases. ^c^ MS(EI): 208(8%), 97(100%), 96(17%), 86(9%), 69(12%), 56(22%), 55(64%), 43(18%).

**Table 5:** Chemical composition of the essential oil of *Polymnia canadensis* L.

RI ^a^	RI ^b^	Compound	% ± SD	RI ^a^	RI ^b^	Compound	% ± SD
802	801	Hexanal	0.28 ± 0.04	1417	1417	β-Caryophyllene	3.05 ± 0.02
811	796	2-Hexanol	0.17 ± 0.01	1428	1430	β-Copaene	0.09 ± 0.02
850	850	(3*Z*)-Hexenol	4.31 ± 0.16	1446	1453	Geranyl acetone	0.17 ± 0.01
861	854	(2*E*)-Hexenol	0.08 ± 0.01	1454	1452	α-Humulene	1.14 ± 0.00
864	863	1-Hexanol	0.30 ± 0.02	1458	1458	*allo*-Aromadendrene	0.17 ± 0.02
921	921	Tricyclene	0.07 ± 0.01	1479	1484	Germacrene D	11.42 ± 0.01
924	924	α-Thujene	0.06 ± 0.01	1484	1486	Phenylethyl 2-methylbutanoate	0.18 ± 0.04
932	932	α-Pinene	19.71 ± 0.11	1487	1489	β-Selinene	0.41 ± 0.03
948	946	Camphene	0.80 ± 0.01	1490	1490	Phenylethyl 3-methylbutanoate	0.09 ± 0.01
971	969	Sabinene	1.96 ± 0.00	1493	1500	Bicyclogermacrene	1.03 ± 0.00
976	974	β-Pinene	0.87 ± 0.01	1496	1500	α-Muurolene	0.12 ± 0.01
987	988	Myrcene	0.53 ± 0.01	1502	1509	Lavandulyl 3-methylbutanoate	0.76 ± 0.01
1006	1002	α-Phellandrene	28.30 ± 0.16	1511	1513	γ-Cadinene	0.21 ± 0.01
1016	1014	α-Terpinene	0.09 ± 0.01	1515	1518	Bornyl 3-methylbutanoate	0.39 ± 0.01
1024	1020	*p*-Cymene	4.42 ± 0.02	1516	1522	δ-Cadinene	0.36 ± 0.01
1028	1024	Limonene	0.38 ± 0.01	1527	1529	Kessane	0.59 ± 0.04
1030	1025	β-Phellandrene	0.06 ± 0.02	1535	1534	Liguloxide	0.84 ± 0.01
1034	1032	(*Z*)-β-Ocimene	0.17 ± 0.01	1559	1561	(*E*)-Nerolidol	1.71 ± 0.01
1044	1044	(*E*)-β-Ocimene	0.19 ± 0.01	1565	1565	Thymyl 2-methylbutanoate	0.69 ± 0.01
1057	1054	γ-Terpinene	0.09 ± 0.01	1568	1570	Neryl 2-methylbutanoate	0.76 ± 0.01
1069	1065	*cis*-Sabinene hydrate	0.08 ± 0.01	1575	1574	Germacrene D-4β-ol	0.18 ± 0.01
1084	1086	Terpinolene	0.07 ± 0.01	1581	1582	Caryophyllene oxide	0.21 ± 0.02
1099	1095	Linalool	tr ^c^	1608	1613	Copaborneol	0.18 ± 0.05
1101	1098	*trans*-Sabinene hydrate	tr	1641	1638	τ-Cadinol	0.59 ± 0.02
1141	1135	*trans*-Pinocarveol	0.08 ± 0.02	1654	1652	α-Cadinol	0.81 ± 0.02
1145	1140	*trans*-Verbenol	0.14 ± 0.01	1657	1658	Selin-11-en-4α-ol	0.15 ± 0.01
1163	1165	Lavandulol	0.12 ± 0.01	1684	1685	Germacra-4(15),5,10(14)-trien-1α-ol	0.49 ± 0.03
1172	1165	Borneol	0.11 ± 0.01	1693	1695	6-*epi*-Shyobunol	0.20 ± 0.02
1180	1174	Terpinen-4-ol	0.26 ± 0.00	2227	d	Kauran-16β-ol	3.48 ± 0.01
1208	1204	Verbenone	0.06 ± 0.01	2243	d	Kauran-16α-ol	0.17 ± 0.02
1228	1232	Thymol methyl ether	2.89 ± 0.01			Green leaf volatiles	5.13
1342	1345	7-*epi*-Silphiperfol-5-ene	0.59 ± 0.02			Monoterpene hydrocarbons	57.78
1351	1356	Eugenol	0.18 ± 0.03			Oxygenated monoterpenoids	6.34
1367	1369	Cyclosativene	0.08 ± 0.01			Sesquiterpene hydrocarbons	18.92
1367	1371	Longicyclene	tr			Oxygenated sesquiterpenoids	5.96
1372	1377	Silphiperol-6-ene	0.06 ± 0.00			Diterpenoids	3.65
1374	1374	α-Copaene	0.12 ± 0.01			Benzenoids	0.45
1380	1382	Modheph-2-ene	0.11 ± 0.00			Others	0.17
1386	1387	β-Cubebene	0.06 ± 0.01			Total Identified	98.40
1387	1389	β-Elemene	0.50 ± 0.01				

^a^ RI = Retention index determined in reference to a homologous series of *n*-alkanes on a ZB-5ms column. ^b^ RI values from the databases. ^c^ tr = “trace” (<0.05%). ^d^ Assignment tentative; based on MS only.

**Table 6:** Chemical composition of the essential oil of *Rudbeckia laciniata* L.

RI ^a^	RI ^b^	Compound	% ± SD	RI ^a^	RI ^b^	Compound	% ± SD
802	801	Hexanal	0.05 ± 0.00	1206	1204	Verbenone	0.10 ± 0.03
810	796	2-Hexanol	0.34 ± 0.01	1218	1215	*trans*-Carveol	0.25 ± 0.06
922	921	Tricyclene	0.10 ± 0.00	1232	1226	*cis*-Carveol	0.07 ± 0.02
924	924	α-Thujene	0.10 ± 0.01	1243	1239	Carvone	0.49 ± 0.02
932	932	α-Pinene	10.18 ± 0.06	1283	1287	Bornyl acetate	2.68 ± 0.02
948	946	Camphene	2.24 ± 0.02	1349	1350	α-Longipinene	0.08 ± 0.02
971	969	Sabinene	0.90 ± 0.01	1368	1369	Cyclosativene	0.06 ± 0.02
977	974	β-Pinene	9.21 ± 0.05	1375	1374	α-Copaene	0.16 ± 0.01
988	988	Myrcene	5.26 ± 0.02	1391	1390	Sativene	0.05 ± 0.01
1004	1003	*p*-Mentha-1(7),8-diene	0.07 ± 0.01	1417	1419	β-Ylangene	tr
1024	1020	*p*-Cymene	0.11 ± 0.01	1418	1417	β-Caryophyllene	0.43 ± 0.03
1029	1024	Limonene	58.07 ± 0.47	1428	1434	γ-Elemene	0.07 ± 0.00
1030	1025	β-Phellandrene	0.74 ± 0.08	1431	1432	*trans*-α-Bergamotene	0.19 ± 0.02
1034	1032	(*Z*)-β-Ocimene	0.09 ± 0.01	1454	1452	α-Humulene	0.12 ± 0.01
1044	1044	(*E*)-β-Ocimene	1.07 ± 0.03	1473	1478	γ-Muurolene	0.05 ± 0.01
1069	1067	*cis*-Linalool oxide (furanoid)	0.19 ± 0.00	1480	1484	Germacrene D	2.52 ± 0.02
1085	1084	*trans*-Linalool oxide (furanoid)	0.05 ± 0.01	1482	1484	(*Z*,*Z*)-α-Farnesene	0.05 ± 0.01
1121	1119	*trans-p*-Mentha-2,8-dien-1-ol	0.37 ± 0.01	1494	1500	Bicyclogermacrene	0.06 ± 0.01
1130	1131	Limona ketone	0.06 ± 0.01	1497	1500	α-Muurolene	0.07 ± 0.01
1132	1132	*cis*-Limonene oxide	0.20 ± 0.00	1514	1514	Cubebol	0.12 ± 0.01
1136	1133	*cis*-p-Mentha-2,8-dien-1-ol	0.26 ± 0.01	1517	1522	δ-Cadinene	0.15 ± 0.01
1136	1137	*trans*-Limonene oxide	0.27 ± 0.01	1575	1574	Germacra-1(10),5-dien-4β-ol	0.20 ± 0.02
1138	1135	Nopinone	0.06 ± 0.01	1581	1582	Caryophyllene oxide	0.19 ± 0.03
1140	1135	*trans*-Pinocarveol	0.19 ± 0.03	1591	1594	Salvial-4(14)-en-1-one	tr
1145	1140	*trans*-Verbenol	0.07 ± 0.01	1601	1594	Carotol	0.18 ± 0.01
1162	1160	Pinocarvone	0.12 ± 0.00	1620	1611	Germacra-1(10),5-dien-4α-ol	0.20 ± 0.01
1171	1165	Borneol	0.11 ± 0.02			Green leaf volatiles	0.39
1178	1179	2-Isopropenyl-5-methyl-4-hexenal	0.08 ± 0.01			Monoterpene hydrocarbons	88.15
1180	1174	Terpinen-4-ol	0.11 ± 0.02			Oxygenated monoterpenoids	6.18
1187	1183	Cryptone	0.10 ± 0.01			Sesquiterpene hydrocarbons	4.06
1195	1195	Myrtenal	0.23 ± 0.02			Oxygenated sesquiterpenoids	0.89
1197	1200	*trans*-Dihydrocarvone	tr ^c^			Total Identified	99.67
1199	1195	*cis*-Piperitol	0.11 ± 0.07				

^a^ RI = Retention index determined in reference to a homologous series of *n*-alkanes on a ZB-5ms column. ^b^ RI values from the databases. ^c^ tr = “trace” (<0.05%).

**Table 7:** Chemical composition of the essential oil of *Silphium integrifolium* Michx.

RI ^a^	RI ^b^	Compound	% ± SD	RI ^a^	RI ^b^	Compound	% ± SD
800	797	(3*Z*)-Hexenal	tr ^c^	1387	1387	β-Cubebene	tr
801	801	Hexanal	0.07 ± 0.02	1388	1389	β-Elemene	0.06 ± 0.01
810	796	2-Hexanol	tr	1417	1419	β-Ylangene	tr
849	846	(2*E*)-Hexenal	0.33 ± 0.02	1418	1417	β-Caryophyllene	2.50 ± 0.02
850	844	(3*E*)-Hexenol	0.27 ± 0.04	1429	1430	β-Copaene	tr
922	921	Tricyclene	0.12 ± 0.00	1432	1432	*trans*-α-Bergamotene	0.13 ± 0.02
925	924	α-Thujene	0.19 ± 0.00	1454	1452	α-Humulene	1.07 ± 0.02
933	932	α-Pinene	58.59 ± 0.21	1469	1471	4,5-di-*epi*-Aristolochene	0.05 ± 0.00
947	945	α-Fenchene	tr	1473	1478	γ-Muurolene	0.06 ± 0.01
949	946	Camphene	2.44 ± 0.02	1480	1484	Germacrene D	2.95 ± 0.01
971	969	Sabinene	1.78 ± 0.00	1482	1484	(*Z*,*Z*)-α-Farnesene	0.10 ± 0.01
977	974	β-Pinene	14.69 ± 0.07	1488	1489	β-Selinene	0.15 ± 0.01
988	988	Myrcene	9.70 ± 0.02	1491	1493	*trans*-Muurola-4(14),5-diene	tr
1004	1003	*p*-Mentha-1(7),8-diene	tr	1494	1500	Bicyclogermacrene	0.08 ± 0.00
1024	1020	*p*-Cymene	tr	1497	1500	α-Muurolene	tr
1028	1024	Limonene	1.76 ± 0.01	1512	1513	γ-Cadinene	tr
1030	1025	β-Phellandrene	0.31 ± 0.03	1517	1522	δ-Cadinene	0.10 ± 0.02
1034	1032	(*Z*)-β-Ocimene	0.05 ± 0.01	1575	1574	Germacra-1(10),5-dien-4β-ol	0.27 ± 0.02
1044	1044	(*E*)-β-Ocimene	0.44 ± 0.03	1581	1582	Caryophyllene oxide	0.47 ± 0.01
1057	1054	γ-Terpinene	tr	1609	1608	Humulene epoxide II	0.13 ± 0.01
1085	1086	Terpinolene	tr	2019	2026	(*E*,*E*)-Geranyl linalool	0.06 ± 0.01
1099	1099	α-Pinene oxide	0.10 ± 0.01	2228	2237	7α-Hydroxymanool	0.16 ± 0.02
1112	1113	(*E*)-4,8-Dimethylnona-1,3,7-triene	tr	2300	2300	Tricosane	tr
1126	1122	α-Campholenal	tr	2500	2500	Pentacosane	0.16 ± 0.01
1140	1135	*trans*-Pinocarveol	tr	2700	2700	Heptacosane	0.16 ± 0.02
1145	1140	*trans*-Verbenol	0.11 ± 0.02			Green leaf volatiles	0.66
1162	1160	Pinocarvone	tr			Monoterpene hydrocarbons	90.09
1180	1174	Terpinen-4-ol	tr			Oxygenated monoterpenoids	0.38
1195	1195	Myrtenal	0.06 ± 0.01			Sesquiterpene hydrocarbons	7.37
1206	1204	Verbenone	0.10 ± 0.02			Oxygenated sesquiterpenoids	1.09
1368	1369	Cyclosativene	0.06 ± 0.00			Others	0.32
1375	1374	α-Copaene	0.08 ± 0.01			Total Identified	99.90
1383	1387	β-Bourbonene	tr				

^a^ RI = Retention index determined in reference to a homologous series of *n*-alkanes on a ZB-5ms column. ^b^ RI values from the databases. ^c^ tr = “trace” (<0.05%).

**Table 8:** Chemical composition of the essential oil of *Smallanthus uvedalia* (L.) Mack.

RI ^a^	RI ^b^	Compound	% ± SD	RI ^a^	RI ^b^	Compound	% ± SD
795	801	2-Methylhept-2-ene	0.10 ± 0.00	1207	1204	Verbenone	0.09 ± 0.01
801	801	Hexanal	0.86 ± 0.16	1346	1345	α-Cubebene	0.15 ± 0.04
850	846	(2*E*)-Hexenal	1.40 ± 0.09	1382	1387	β-Bourbonene	0.15 ± 0.02
865	863	1-Hexanol	0.22 ± 0.01	1418	1417	β-Caryophyllene	3.80 ± 0.07
922	921	Tricyclene	0.08 ± 0.00	1454	1452	α-Humulene	0.36 ± 0.02
924	924	α-Thujene	1.28 ± 0.02	1473	1478	γ-Muurolene	0.56 ± 0.12
932	932	α-Pinene	62.56 ± 0.79	1512	1513	γ-Cadinene	0.29 ± 0.09
948	946	Camphene	1.35 ± 0.01	1517	1522	δ-Cadinene	0.63 ± 0.03
971	969	Sabinene	0.16 ± 0.03	1536	1537	α-Cadinene	0.22 ± 0.04
977	974	β-Pinene	6.00 ± 0.09	1576	1577	Spathulenol	0.58 ± 0.11
988	988	Myrcene	2.43 ± 0.07	1581	1582	Caryophyllene oxide	1.37 ± 0.02
1024	1020	*p*-Cymene	0.15 ± 0.01			Green leaf volatiles	2.48
1028	1024	Limonene	11.43 ± 0.11			Monoterpene hydrocarbons	88.28
1030	1025	β-Phellandrene	0.70 ± 0.10			Oxygenated monoterpenoids	0.74
1044	1044	(*E*)-β-Ocimene	1.87 ± 0.09			Sesquiterpene hydrocarbons	6.16
1057	1054	γ-Terpinene	0.26 ± 0.01			Oxygenated sesquiterpenoids	1.95
1126	1122	α-Campholenal	0.29 ± 0.01			Others	0.10
1140	1135	*trans*-Pinocarveol	0.23 ± 0.04			Total Identified	99.71
1145	1140	*trans*-Verbenol	0.14 ± 0.05			

^a^ RI = Retention index determined in reference to a homologous series of *n*-alkanes on a ZB-5ms column. ^b^ RI values from the databases.

**Table 9:** Chemical composition of the essential oil of *Solidago altissima* L.

RI ^a^	RI ^b^	Compound	% ± SD	RI ^a^	RI ^b^	Compound	% ± SD
802	801	Hexanal	0.17 ± 0.01	1382	1387	β-Bourbonene	tr
850	846	(2*E*)-Hexenal	1.21 ± 0.03	1386	1387	β-Cubebene	0.10 ± 0.01
921	921	Tricyclene	0.06 ± 0.00	1387	1389	β-Elemene	0.18 ± 0.00
924	924	α-Thujene	1.30 ± 0.00	1416	1419	β-Ylangene	0.14 ± 0.01
931	932	α-Pinene	13.91 ± 0.04	1418	1417	β-Caryophyllene	0.50 ± 0.03
948	946	Camphene	2.41 ± 0.01	1428	1430	β-Copaene	0.14 ± 0.01
971	969	Sabinene	14.25 ± 0.03	1454	1452	α-Humulene	0.17 ± 0.00
976	974	β-Pinene	4.62 ± 0.02	1473	1478	γ-Muurolene	0.59 ± 0.03
988	988	Myrcene	20.29 ± 0.04	1477	1483	α-Amorphene	0.12 ± 0.02
1005	1004	(3*Z*)-Hexenyl acetate	tr ^c^	1479	1484	Germacrene D	10.67 ± 0.03
1006	1002	α-Phellandrene	2.84 ± 0.02	1487	1489	β-Selinene	tr
1016	1014	α-Terpinene	0.10 ± 0.00	1490	1495	γ-Amorphene	0.59 ± 0.01
1024	1020	*p*-Cymene	2.26 ± 0.00	1494	1500	Bicyclogermacrene	0.18 ± 0.00
1028	1024	Limonene	1.27 ± 0.01	1496	1500	α-Muurolene	0.14 ± 0.01
1030	1025	β-Phellandrene	0.35 ± 0.01	1511	1513	γ-Cadinene	0.30 ± 0.01
1044	1044	(*E*)-β-Ocimene	0.08 ± 0.01	1513	1514	Cubebol	0.06 ± 0.02
1057	1054	γ-Terpinene	0.38 ± 0.00	1516	1522	δ-Cadinene	0.68 ± 0.02
1069	1065	*cis*-Sabinene hydrate	0.24 ± 0.00	1535	1537	α-Cadinene	0.09 ± 0.01
1084	1086	Terpinolene	0.15 ± 0.01	1547	1548	Elemol	0.06 ± 0.01
1090	1090	6,7-Epoxymyrcene	0.05 ± 0.00	1575	1574	Germacra-1(10),5-dien-4β-ol	0.14 ± 0.01
1099	1099	α-Pinene oxide	tr	1581	1582	Caryophyllene oxide	0.06 ± 0.01
1101	1098	*trans*-Sabinene hydrate	0.15 ± 0.00	1591	1594	Salvial-4(14)-en-1-one	0.06 ± 0.01
1105	1100	Nonanal	tr	1619	1611	Germacra-1(10),5-dien-4α-ol	0.12 ± 0.01
1112	1113	(*E*)-4,8-Dimethylnona-1,3,7-triene	0.11 ± 0.03	1627	1629	*iso*-Spathulenol	0.20 ± 0.01
1124	1124	*cis-p*-Menth-2-en-1-ol	0.05 ± 0.00	1641	1638	τ-Cadinol	0.09 ± 0.02
1180	1174	Terpinen-4-ol	0.73 ± 0.01	1643	1640	τ-Murrolol	0.14 ± 0.01
1195	1186	α-Terpineol	0.06 ± 0.01	1645	1644	α-Muurolol (=δ-Cadinol)	0.10 ± 0.01
1203	1202	*cis*-Sabinol	0.50 ± 0.01	1654	1652	α-Cadinol	0.41 ± 0.03
1219	1219	*cis*-Sabinene hydrate acetate	0.15 ± 0.01			Green leaf volatiles	1.38
1283	1287	Bornyl acetate	14.44 ± 0.02			Monoterpene hydrocarbons	64.26
1333	1335	δ-Elemene	0.05 ± 0.00			Oxygenated monoterpenoids	16.37
1345	1345	α-Cubebene	0.12 ± 0.00			Sesquiterpene hydrocarbons	14.90
1367	1373	α-Ylangene	tr			Oxygenated sesquiterpenoids	1.44
1368	1373	Linalyl isobutyrate	tr			Others	0.11
1374	1374	α-Copaene	0.05 ± 0.00			Total Identified	98.45

^a^ RI = Retention index determined in reference to a homologous series of *n*-alkanes on a ZB-5ms column. ^b^ RI values from the databases. ^c^ tr = “trace” (<0.05%).

**Table 10:** Comparison of the major components in *Solidago altissima* (syn. *S. canadensis*) essential oils.

Component	Source of *S. altissima* (*S. canadensis*)									
	Commercial (Young Living) [85]	Bimtal, India [86]	Bimtal, India [87]	Slovakia [88]	Moscow, Russia [89]	Slovakia [90]	Hungary [91]	Giza, Egypt [92]	Poland [82]	Alabama (This Work)
α-Pinene	13.3	5.0	0.4	1.8–36.3	28.1	11.6	4.6	29.2	14.7	13.9
Sabinene	8.0	2.4	0.3	---	0.5	3.9	0.1	---	0.2	14.2
β-Pinene	---	1.2	0.2	0.5–6.5	2.8	3.1	1.2	4.8	1.5	4.6
Myrcene	6.3	2.8	---	---	7.3	---	tr	13.7	4.2	20.2
Limonene	11.0	12.5	4.2	4.3-9.0	7.0	12.5	1.0	9.6	9.3	1.3
Bornyl acetate	4.3	2.1	3.4	---	7.3	6.3	13.4	6.2	1.3	14.4
Germacrene D	34.4	56.7	64.1	0.0–11.1	39.2	34.9	11.0	10.3	19.8	10.6

**Table 11:** Chemical composition of the essential oil of *Xanthium strumarium* L.

RI ^a^	RI ^b^	Compound	% ± SD	RI ^a^	RI ^b^	Compound	% ± SD
793	788	1-Octene	0.09 ± 0.01	1417	1417	β-Caryophyllene	0.93 ± 0.04
801	797	(3*Z*)-Hexenal	0.11 ± 0.01	1428	1430	β-Copaene	0.06 ± 0.01
802	801	Hexanal	0.75 ± 0.07	1454	1452	α-Humulene	0.49 ± 0.03
859	846	(2*E*)-Hexenal	5.79 ± 0.03	1479	1484	Germacrene D	13.92 ± 0.05
865	863	1-Hexanol	0.12 ± 0.01	1487	1489	β-Selinene	0.11 ± 0.01
921	921	Tricyclene	0.05 ± 0.01	1493	1500	Bicyclogermacrene	0.29 ± 0.01
924	924	α-Thujene	0.08 ± 0.03	1496	1500	α-Muurolene	0.13 ± 0.01
931	932	α-Pinene	0.80 ± 0.01	1511	1513	γ-Cadinene	0.19 ± 0.01
948	946	Camphene	0.95 ± 0.02	1516	1522	δ-Cadinene	0.27 ± 0.03
971	969	Sabinene	4.89 ± 0.02	1575	1547	Germacra-1(10),5-dien-4β-ol	0.21 ± 0.01
976	974	β-Pinene	0.30 ± 0.01	1581	1582	Caryophyllene oxide	0.23 ± 0.01
978	974	1-Octen-3-ol	0.20 ± 0.02	1641	1638	τ-Cadinol	0.31 ± 0.02
987	988	Myrcene	14.31 ± 0.04	1643	1640	τ-Muurolol	0.23 ± 0.02
1004	1003	*p*-Mentha-1(7),8-diene	0.05 ± 0.01	1654	1652	α-Cadinol	0.59 ± 0.05
1016	1014	α-Terpinene	0.06 ± 0.01	1663	1668	*ar*-Turmerone	0.10 ± 0.01
1028	1024	Limonene	48.23 ± 0.22	1693	1688	Shyobunol	0.27 ± 0.03
1030	1025	β-Phellandrene	0.90 ± 0.03	1932	1931	Beyerene	0.64 ± 0.02
1044	1044	(*E*)-β-Ocimene	0.10 ± 0.02	2105	2106	(*E*)-Phytol	0.25 ± 0.03
1057	1054	γ-Terpinene	0.16 ± 0.01			Green leaf volatiles	6.77
1069	1067	*cis*-Linalool oxide (furanoid)	0.06 ± 0.01			Monoterpene hydrocarbons	70.87
1099	1095	Linalool	1.16 ± 0.01			Oxygenated monoterpenoids	2.22
1180	1174	Terpinen-4-ol	0.39 ± 0.01			Sesquiterpene hydrocarbons	16.58
1219	1217	β-Cyclocitral	0.11 ± 0.02			Oxygenated sesquiterpenoids	1.95
1283	1287	Bornyl acetate	0.56 ± 0.01			Diterpenoids	0.89
1351	1356	Eugenol	0.28 ± 0.03			Benzenoids	0.28
1386	1387	β-Cubebene	0.10 ± 0.02			Others	0.29
1416	1419	β-Ylangene	0.08 ± 0.03			Total Identified	99.85

^a^ RI = Retention index determined in reference to a homologous series of *n*-alkanes on a ZB-5ms column. ^b^ RI values from the databases.

**Table 12:** Major components and antifungal activities of Asteraceae essential oils.

Plant Species	Major Components (>5%) in the Essential Oil	Antifungal Activity, MIC, μg/mL ^a^		
		*Aspergillus niger*	*Candida albicans*	*Cryptococcus neoformans*
*Eupatorium serotinum* Michx.	germacrene D (6.6%), palustrol (5.4%), cyclocolorenone (23.5%)	313	625	78
*Eurybia macrophylla* (L.) Cass.	β-pinene (8.5%), limonene (28.6%), terpinolene (5.3%), germacrene D (19.7%), germacrene B (7.0%)	625	625	156
*Eutrochium purpureum* (L.) E.E. Lamont	hexanal (6.8%), (2*E*)-hexenal (59.7%), methyl salicylate (10.4%), eugenol (11.8%)	625	625	625
*Polymnia canadensis* L.	α-pinene (19.6%), α-phellandrene (28.2%), germacrene D (11.4%)	625	625	156
*Rudbeckia laciniata* L.	α-pinene (10.2%), β-pinene (9.2%), myrcene (5.3%), limonene (58.9%)	625	1250	156
*Silphium integrifolium* Michx.	α-pinene (58.5%), β-pinene (14.7%), myrcene (9.7%)	n.t. ^b^	n.t.	n.t.
*Smallanthus uvedalia* (L.) Mack.	α-pinene (62.3%), β-pinene (6.0%), limonene (11.3%)	n.t.	n.t.	n.t.
*Solidago altissima* L.	α-pinene (13.9%), sabinene (14.2%), myrcene (20.2%), bornyl acetate (14.4%), germacrene D (10.6%)	625	1250	313
*Xanthium strumarium* L.	(2*E*)-hexenal (5.8%), myrcene (14.3%), limonene (48.0%), germacrene D (13.9%)	625	1250	n.t.

^a^ Each minimum inhibitory concentration (MIC) determination was carried out in triplicate. ^b^ n.t. = not tested due to limited availability of the essential oil.

**Table 13:** Antifungal activities (MIC, μg/mL) of essential oil components.

Compound	*Aspergillus niger*	*Candida albicans*	*Cryptococcus neoformans*
α-Pinene	1250	625	313
β-Pinene	625	1250	625
Limonene	625	1250	625
Myrcene	625	625	625
Methyl salicylate	625	625	625
Eugenol	78	313	156
Bornyl acetate	625	625	625

**Table 14:** Plant collection sites and essential oil yields of Asteraceae from north Alabama.

Plant	Collection Site, Date	Voucher Number	Mass of Aerial Parts (g)	Yield of Essential Oil (mg)
*Eupatorium serotinum* Michx.	34°38′29″ N, 86°24′39″ W, elev. 199 m 13 September 2018	233754	49.09	6.4 (0.013%)
*Eurybia macrophylla* (L.) Cass.	34°39′25″ N, 86°24′45″ W, elev. 241 m 15 September 2018	233117	56.56	10.6 (0.019%)
*Eutrochium purpureum* (L.) E.E. Lamont	34°38′40″ N, 86°27′22″ W, elev. 180 m 12 August 2018	091843	63.44	12.3 (0.019%)
*Polymnia canadensis* L.	34°38′29″ N, 86°24′39″ W, elev. 199 m 21 July 2018	184700	52.89	39.1 (0.074%)
*Rudbeckia laciniata* L.	34°42′42″ N, 86°32′38″ W, elev. 345 m 13 September 2018	004426	54.53	6.0 (0.011%)
*Silphium integrifolium* Michx.	34°42′42″ N, 86°32′38″ W, elev. 345 m 15 September 2018	004152	15.02	6.4 (0.043%)
*Smallanthus uvedalia* (L.) Mack.	34°42′42″ N, 86°32′38″ W, elev. 345 m 15 September 2018	000714	56.21	5.9 (0.010%)
*Solidago altissima* L.	34°38′40″ N, 86°27′22″ W, elev. 180 m 12 August 2018	001425	54.41	44.9 (0.083%)
*Xanthium strumarium* L.	34°38′49″ N, 86°24′38″ W, elev. 200 m 15 September 2018	224724	69.69	7.0 (0.010%)
